# Effects of an eight-week team handball intervention on balance and leg strength in older adults: a pilot study

**DOI:** 10.3389/fspor.2026.1811953

**Published:** 2026-07-31

**Authors:** Janik Dürselen, Stefanie Klatt, Frowin Fasold

**Affiliations:** Institute of Exercise Training and Sport Informatics, Section II: Cognition in Team Sports, German Sport University Cologne, Cologne, Germany

**Keywords:** fall prevention, functional mobility, functional performance, multidimensional balance, neuromuscular adaptations, physical inactivity, small-sided games

## Abstract

**Introduction:**

With increasing age, functional decline leads to reduced physical activity and an elevated risk of falls. Positive effects on musculoskeletal, metabolic, and cardiovascular systems have already been observed from recreational team sports. This study, therefore, aimed to investigate the effects of team handball-based activities on balance and leg strength in inactive, healthy older adults. We hypothesized that the intervention would improve balance and leg strength.

**Method:**

In this quasi-experimental pilot study participants were assigned to a team handball group (HG; *n* = 7; 72.57 ± 5.44 years) or a control group (CG; *n* = 6; 66.83 ± 2.64 years). HG conducted an eight-week small-sided team handball-based intervention (twice-weekly, 60 min), while the control group performed their usual activity. Static steady-state, dynamic steady-state, proactive, reactive balance, and leg strength were assessed at pre-, post-, and retention-testing (six weeks post-intervention).

**Results:**

Repeated-measures ANOVA (rmANOVA) revealed no significant group × time interaction effects. Where the assumptions for rmANOVA were violated, Mann–Whitney U tests revealed a between-group difference in change scores for static steady-state balance (eyes open) from pre- to retention-test (*p* = .032), with the HG showing greater improvement than the CG. Significant within-group effects of time were observed in the HG for proactive balance (*p* = .018) and leg strength (*p* = .021). post hoc analyses revealed a significant decline in proactive balance from post- to retention-test (*p* = .023) and significant improvements in leg strength from pre- to post-test (*p* = .048) and from pre- to retention-test (*p* = .048). No other significant between-group or within-group effects were observed.

**Discussion & conclusion:**

Modified team handball-based activities may represent a feasible, enjoyable, and engaging form of physical activity for older adults. The significant between-group difference observed at the retention-test may suggest a delayed training effect; however, these findings should be interpreted cautiously given the exploratory nature of this pilot study. Short-term participation in a structured team handball-based activity program was associated with improved leg strength within the intervention group. Balance outcomes did not show significant within-group improvements, and the decline in proactive balance at the retention-test may indicate a potential detraining effect, indicating that longer intervention periods may be required. Overall, modified team handball-based activities may represent a valuable complement to traditional exercise programs for older adults.

## 1 Introduction

In recent years, scientific research has frequently focused on demographic change and the associated challenges for society (1). As life expectancy rises and the population ages, the risk of health problems increases (2, 3). Maintaining independence and functional capacity in old age is therefore crucial for ensuring quality of life. Over 60% of older adults (aged 65 and over) report having at least one chronic disease or long-term health problem (1, 2).

Among age-related health concerns, falls represent one of the most critical threats to autonomy and well-being in later life (4). Up to 40% of older adults experience at least one fall each year, which can result in moderate to severe injuries, fear of falling, loss of independence, reduced quality of life, and even death (5–7). Impaired balance and reduced muscle strength are associated with an increased risk of falling and a greater fear of falling (8). Together, an increased fear of falling and functional limitations can lead to social withdrawal and avoidance of everyday activities in older adults (9). Accordingly, exercise interventions targeting both physical function and fear of falling are of particular relevance (10). Such interventions simultaneously target psychological and physiological determinants of fall risk, thereby addressing both the cause and the consequence of reduced functional capacity.

Balance and muscle strength are key targets that can be improved through exercise (11). Research has identified well-established exercise programs that significantly reduce the risk of falling. Numerous studies have demonstrated the effectiveness of strength, flexibility, and balance training in reducing the risk of falls and improving physical function (12–14). However, adherence to these traditional exercise programs often declines over time due to monotony and lack of social engagement, limiting their long-term effectiveness (15).

In addition to these conventional approaches, the health-promoting effects of team sports activities on metabolic and cardiovascular systems have been investigated [e.g. (16),]. These activities expand the range of traditional health-oriented exercise programs and provide older adults with an appealing, socially engaging, and cognitively stimulating opportunity to re-engage in structured physical activity (15). Moreover, the social and competitive elements inherent in team sports may enhance motivation, enjoyment, and long-term adherence among older adults.

Evidence suggests that soccer- and team handball-based activities, next to the beneficial effect on the metabolic and cardiovascular system, can improve balance and reduce fall risk indicators (17, 18). However, most of this evidence originates from studies with younger or healthy adults, while data on older populations at increased fall risk remain scarce (18). Thus, investigating the applicability of team handball-based activities in older populations could provide valuable insights into the potential of team sports-based activities for fall prevention. From a physiological and neuromuscular perspective, the multidirectional movements, reactive demands, and intermittent load patterns of team handball may provide specific stimuli that enhance balance control and leg strength. Both are critical for fall prevention.

Team sports are typically characterized by high intermittent intensities and are often implemented as time-efficient recreational formats, with exercise sessions lasting around 60 min (15). Recreational game formats are regularly organized as small-sided games (e.g., three vs. three, four vs. four, or five vs. five) (15, 19). Due to the combined aerobic and anaerobic demands of intermittent exercises, training loads in recreational team sports are similar to those in competitive team sports and average between 72% and 85% of maximum heart rate (19). This range is known to maintain and improve cardiovascular fitness (15). In particular recreational soccer (20, 21), team handball (16), and volleyball (22), have consistently been shown to improve cardiovascular and metabolic parameters in both younger adults and older populations. Furthermore, studies examining 12- to 16-week soccer-, team handball-, and basketball-based activities have demonstrated an increase in maximal oxygen uptake across various age groups, including middle-aged and older adults (16, 21, 23, 24). Typical intervention durations range from eight to 16 weeks, including one to three sessions per week, suggesting that even relatively short programs can induce measurable cardiovascular and neuromuscular adaptations in older adults (15).

Overall, previous studies indicate that team sport-based activities can positively affect the cardiovascular, metabolic and musculoskeletal systems [for review (15, 25),]. The latter is particularly relevant for balance and fall prevention in older adults. However, research on balance and leg strength outcomes in team sports, especially team handball, is lacking in older populations. Given these gaps, further research is needed to explore whether the specific movement and cognitive demands of small-sided team handball can translate into improved fall-related functional outcomes in older adults.

Therefore, the present study aims to investigate the effects of small-sided team handball-based activities on balance and leg strength in older adults.

## 2 Materials and methods

### 2.1 Sample

A total of 14 participants were assigned to either the team handball-based intervention group (HG) or the control group (CG). Group allocation was conducted on a voluntary basis without randomization, as participants could decide, based on personal interest, which group to join. Group self-selection was permitted given the exploratory nature of this pilot study and to maintain participants' motivation and compliance. Exclusion criteria comprised regular participation in other (organized) sports programs (e.g., club sports), health-related limitations (e.g., physical or psychological), mobility restrictions (e.g., use of assistive devices), and prolonged absence. At the time of recruitment, all participants were physically inactive and did not engage in any structured sports. Physical inactivity was an explicit inclusion criterion. During the intervention period, participants in the control group were instructed to maintain their usual daily physical activity levels and were advised not to participate in any additional organized sporting activities. The study was approved by the Research Ethics Committee of the German Sport University Cologne (Ethics application no. 122/2022) and was conducted in accordance with the Declaration of Helsinki. All participants provided written informed consent prior to study participation. Throughout the study period, participants had the right to withdraw from the intervention or the study at any time without providing reasons.

During the intervention, one participant discontinued participation for health reasons unrelated to the study protocol. Consequently, data from 13 participants were included in the final analysis: seven in the HG (*m* = 5, f = 2, age = 72.57 ± 5.44 years, height = 1.76 ± 0.07 m, weight = 90.86 ± 13.43 kg) and six in the CG (m = 4, f = 2, age = 66.83 ± 2.64 years, height = 1.74 ± 0.09 m, weight = 88.00 ± 12.28 kg).

### 2.2 Design

**Figure 1:**
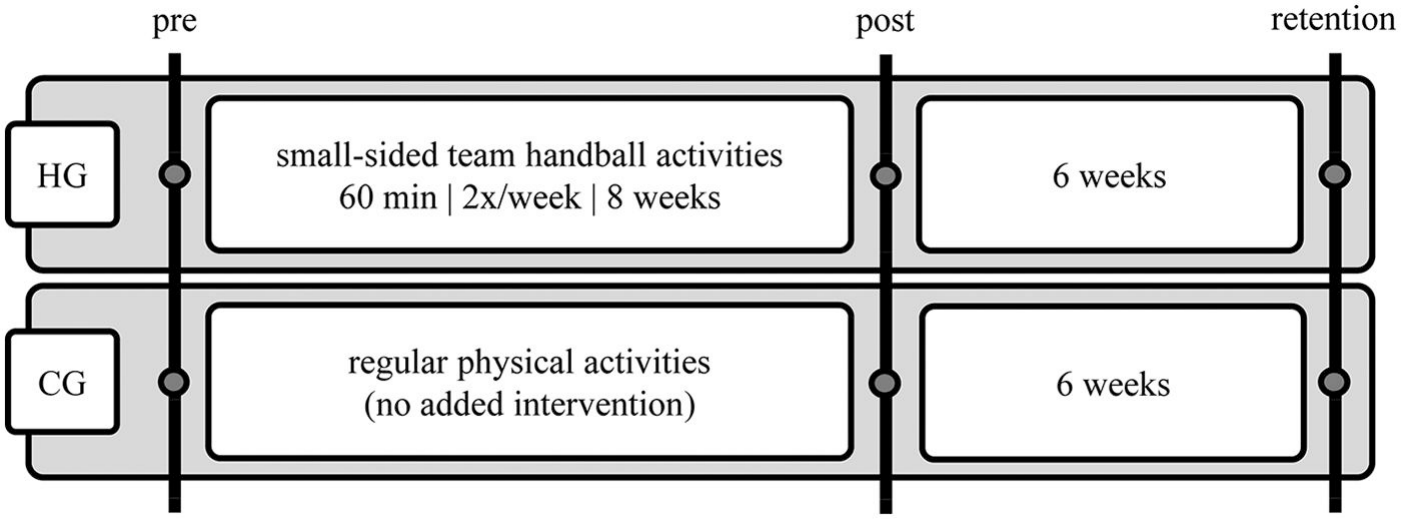
Schematic representation of study design, procedures and measurement time points. min = minutes; pre, post, retentions = measurement time points.

This exploratory, quasi-experimental pilot study was conducted over eight weeks. The HG performed a team handball-based intervention adapted to the target group, while the CG maintained their usual physical activity routines without additional intervention. Balance and leg strength were assessed using a pre-post design with an additional retention-test six weeks after the intervention (Figure 1). All measurements were performed under standardized conditions by the same investigator to minimize measurement bias. Thus, the effect of the between subject-factor *group* (HG vs. CG), and the within-subject factor *measurement time* (pre- vs. post- vs. retention-test) on the *performance outcome* in the different tests was evaluated.

### 2.3 Procedures

To obtain a detailed overview of balance adaptations achieved through the intervention, balance was tested in a task-specific and comprehensive manner. This approach was based on the methods described by Granacher et al. (26), with a focus on the following types of balance: static steady-state, dynamic steady-state, proactive, and reactive. Balance was assessed before leg strength to avoid potential fatigue-related interference.

The team handball-based intervention lasted eight weeks, with participants attending two 60 min sessions per week. A minimum interval of 48 h between sessions was ensured to allow sufficient musculoskeletal recovery. Sessions were conducted by a qualified instructor, experienced in team handball coaching, a team handball coaching license B, and a degree in sports science. Each session included a warm-up phase to slowly prepare the participants mentally and physically to the upcoming exertion of team handball-based activities (approx. 15 min/session). The proceeding 45 min focused on learning and practicing a team handball-based small-sided game: five-a-side handball. This game format, promoted by the German Handball Federation (DHB), includes ten rule modifications compared to regular team handball (27). It is designed for all age groups, skill levels, and genders. Key elements include a playing field measuring 26 × 20 m, a soft foam ball and a strict no-contact rule. In this study, the rules were adapted to the participants' skill levels. Since there were no substitutes available, the format was adjusted to 4-on-4. If there was an odd number of participants, an external player (not a study participant) was added to one team. The no-contact rule was strictly enforced and actively monitored by the coach, with the game being interrupted as needed. Across all sessions, attention was paid to progressively increase load and complexity to familiarize the participants with the sport of team handball and the physical activity. During the first two weeks of the intervention, the participants were gradually introduced to the sport of team handball so that handball-specific movements (e.g., jogging, jumping, landing, stopping, and change of direction) could be developed slowly and in a safe environment. During this phase, all activities were performed at a walking pace in order to minimize the risk of injury and enable participants to safely familiarize themselves with handball-specific movement patterns. In weeks three to eight of the intervention, the physical and technical demands were gradually increased with each exercise session. From week three onwards, participants were allowed and encouraged to start jogging. The sessions included a series of exercises and games that contributed to the development of basic skills and abilities in team handball. Throughout the intervention, all participants demonstrated the ability to catch, pass, and throw the soft, squishy ball. In each session participants were randomly reassigned to new teams to promote social interaction and engagement. The intervention emphasized a game-based approach to team handball.

While the HG conducted the intervention, the CG continued with their usual daily routine. After this eight-week period, the testing was repeated in the post-test, followed by a retention-test six weeks later.

### 2.4 Measurements

*Static steady-state balance* was assessed in terms of standing stability using a modification of the Romberg test (26, 28). For this test, the participants stand on one leg on an unstable surface (50 × 40 × 6 cm). The test is performed twice with eyes open and twice with eyes closed. The arms are held at the hips throughout the test. The time that can be maintained in the one-legged position is measured with the longest standing time in each category taken for analyses. If there is a change in position (e.g., foot position, arm position, etc.), the test is stopped (29).

The *dynamic steady-state balance* was determined considering gait stability using the 10 m walkway test (26, 28, 30). For this test, the participants walk a straight distance of 10 m at their usual speed. The time required to cover a distance of 6 m is measured and reflects the participant's preferred walking speed.

The timed up & go test was performed to assess *proactive balance*, taking mobility into account (26, 28). In this test, the participants perform a combination of standing up from a chair, walking 3 m, turning around, walking back, and sitting down. The time required to complete the entire sequence is measured. The test is performed once.

In addition, *reactive balance* was assessed using the push & release test to evaluate balance recovery (26, 28). In this test, the subjects react to a disruptive stimulus. To do this, the participants first press their shoulders against the hands of the investigator, who is standing behind them. As soon as the pressure is built up and the shoulders fall directly behind the heels, the investigator releases their grip, which represents the disturbance stimulus. The participants are required to regain their balance as quickly as possible. The steps necessary to regain balance are recorded. The test performance of the participants is rated on a 0–4 scale: 0 corresponds to a compensatory step without assistance at normal stride length and width, 1 corresponds to two to three short compensatory steps without assistance, 2 corresponds to four or more lunges without assistance, 3 corresponds to several compensatory steps with assistance to prevent a fall, and 4 corresponds to an immediate fall without compensatory steps, which can only be prevented with assistance (26, 31). The test is repeated three times in total and the best result is used for analysis.

*Leg strength* was measured using the chair rise test (26). The participants stand up from a chair five times as quickly and confidently as possible and then sit back down. The time required to complete a total of five repetitions is measured (26).

All measurement instruments have demonstrated high test–retest reliability and validity in older populations (mod. Romberg test: *r* = 0.99; 10 m walkway test: ICC = 0.96; timed up & go test: *r* = 0.99; push & release test: ICC = 0.84; chair rise test: ICC = 0.89) (31–35).

### 2.5 Statistical analysis

Data are presented as mean and standard deviation (M ± SD). Between-group differences were analyzed using a two-factor (group × time), repeated-measures ANOVA (rmANOVA) for all variables. Prior to analysis, the normality of the residuals was assessed using the Shapiro–Wilk test and the sphericity was evaluated using the Mauchly test. When sphericity was violated, the Greenhouse-Geisser correction was applied. When significant interaction effects were observed, simple main effects were subsequently calculated. Effect sizes were calculated using partial eta squared (*η*_p_^2^) and interpreted according to Cohen (36). Given the small sample size and potential violations of normality, the results of the rmANOVA should be interpreted with caution, as the robustness of parametric tests may be limited under these conditions. However, the rmANOVA was retained for between-group comparisons because it is the most widely accepted approach for analyzing group × time interactions in exercise intervention research and is considered relatively robust against moderate violations of normality.

Where rmANOVA assumptions were violated, Mann–Whitney U tests were conducted as the primary between-group analysis on change scores (*Δ*). Change scores were calculated as the difference between two measurement time points for each participant individually: pre- to post-test (*Δ*1), post- to retention-test (*Δ*2), and pre- to retention-test (*Δ*3). Effect sizes were calculated as *r* and interpreted according to Cohen (36).

Within-group effects over time were analyzed using Friedman test for repeated measures across three time points (pre, post, retention) for all dependent variables. Due to the small sample size, a nonparametric Friedman test was applied, as this test does not require the dependent variable be normally distributed and is therefore more appropriate for small samples. The two-factor rmANOVA was retained for between-subject comparisons because it is the most widely accepted and established approach for analyzing group × time interactions in exercise intervention research. Significant main effects were further examined with Bonferroni-adjusted post hoc tests (i.e., pair-wise comparisons). Effect sizes were calculated using Kendall's W (*W*) and interpreted according to Cohen (36).

## 3 Results

In the following, the results of all measurements at three time points are shown for the HG and CG. The results are presented based on task-specific instruments of balance and leg strength. First, the results of the between-subject factor are presented, followed by the results of the within-subject factor for each group. Table 1 provides descriptive statistics (means and standard deviations) for all outcome measures across all three time points.

**Table 1:** Descriptive statistics for all outcome measures at pre-, post-, and retention-test for the HG and CG.

Measurements	Group	Pre		Post		Retention	
		M	SD	M	SD	M	SD
Mod. Romberg test (eo) [s]	HG	4.85	2.73	6.44	5.92	9.39	7.42
	CG	17.22	14.05	14.94	12.21	13.98	12.68
Mod. Romberg test (ec) [s]	HG	2.44	1.08	2.90	0.83	3.27	1.06
	CG	3.13	1.78	2.96	1.43	3.06	0.91
10 m walkway test [m/s]	HG	1.48	0.19	1.52	0.12	1.58	0.23
	CG	1.43	0.16	1.39	0.18	1.42	0.12
Timed up & go test [s]	HG	7.27	1.39	6.74	1.13	7.43	1.14
	CG	7.60	0.98	7.29	0.77	7.32	0.60
Push & release test [1–4]	HG	2.14	0.69	1.57	0.53	1.86	0.38
	CG	2.33	0.82	2.00	0.63	2.00	0.63
Chair rise test [s]	HG	12.83	1.23	10.65	2.31	11.15	1.65
	CG	11.38	1.59	10.91	1.09	11.60	1.82

eo, eyes open; ec, eyes closed; M, mean; SD, standard deviation; HG, handball group; CG, control group; Pre, pre-test; Post, post-test; Retention, retention-test.

### 3.1 Static steady-state balance

**Figure 2:**
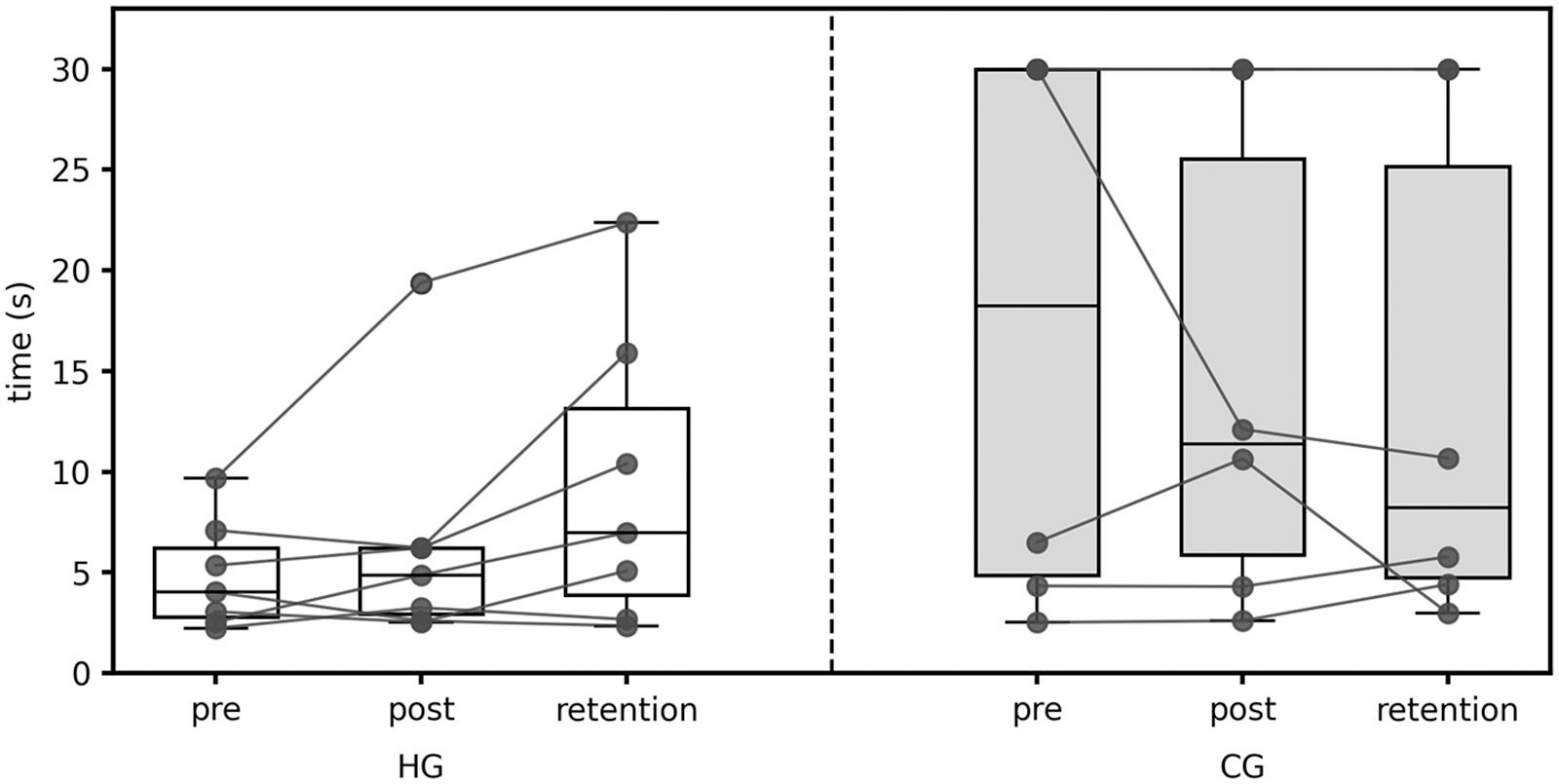
Box plots and intra-individual trends of mod. Romberg test with eyes open at the measurement points (pre, post, and retention), separated by HG and CG. Note. s = seconds; horizontal line = median; box = interquartile range; dispersion of values via whiskers; intra-individual trends are shown by connecting the measured values per person at the three measurement points within the respective group.

Although the modified Romberg test with eyes open apparently shows differences between the groups in the descriptive data, they could not be statistically verified due to the non-normal distribution of the data. However, Mann–Whitney U tests were applied to the change scores (see Table 2 for descriptive statistics) There were no statistically significant between-group differences observed from pre- to post-test (*Δ*1: *U* = 18.00, *z* = −.429, *p* = .668, *r* = .12) or from post- to retention-test (*Δ*2: *U* = 8.00, *z* = −1.860, *p* = .063, *r* = .52). However, a statistically significant difference between the groups was observed from the pre- to retention-test (*Δ*3: *U* = 6.00, *z* = −2.146, *p* = .032, *r* = .60), indicating greater improvement in the HG than in the CG (HG: median *Δ* = 3.31 s; CG: median *Δ* = .00 s). No significant time effects were observed for single-leg stance performance in the HG, *χ*^2^(2) = 3.71, *p* = .156, *W* = .27, or CG, *χ*^2^(2) = 0.00, *p* = 1.00, *W* = .00 (Figure 2).

**Table 2:** Median change scores (*Δ*) for between-group comparisons by group and interval.

Variable	Group	*Δ*1 Mdn (IQR)	*Δ*2 Mdn (IQR)	*Δ*3 Mdn (IQR)
Mod. Romberg test (eo) [s]	HG	0.87 (2.37)	2.55 (2.66)	3.31 (6.25)
	CG	0.00 (0.08)	0.00 (2.18)	0.00 (3.72)
Push & release test [1–4]	HG	0 (1.5)	0 (0.5)	0 (1)
	CG	0 (0.75)	0 (0)	−0.5 (1)

Mdn, Median; IQR, Interquartile Range; HG, handball group; CG, control group; eo, eyes open; *Δ*1, pre- to post-test change score; *Δ*2, post- to retention-test change score; *Δ*3, pre- to retention-test change score. Positive values indicate improvement; negative values indicate decline.

**Figure 3:**
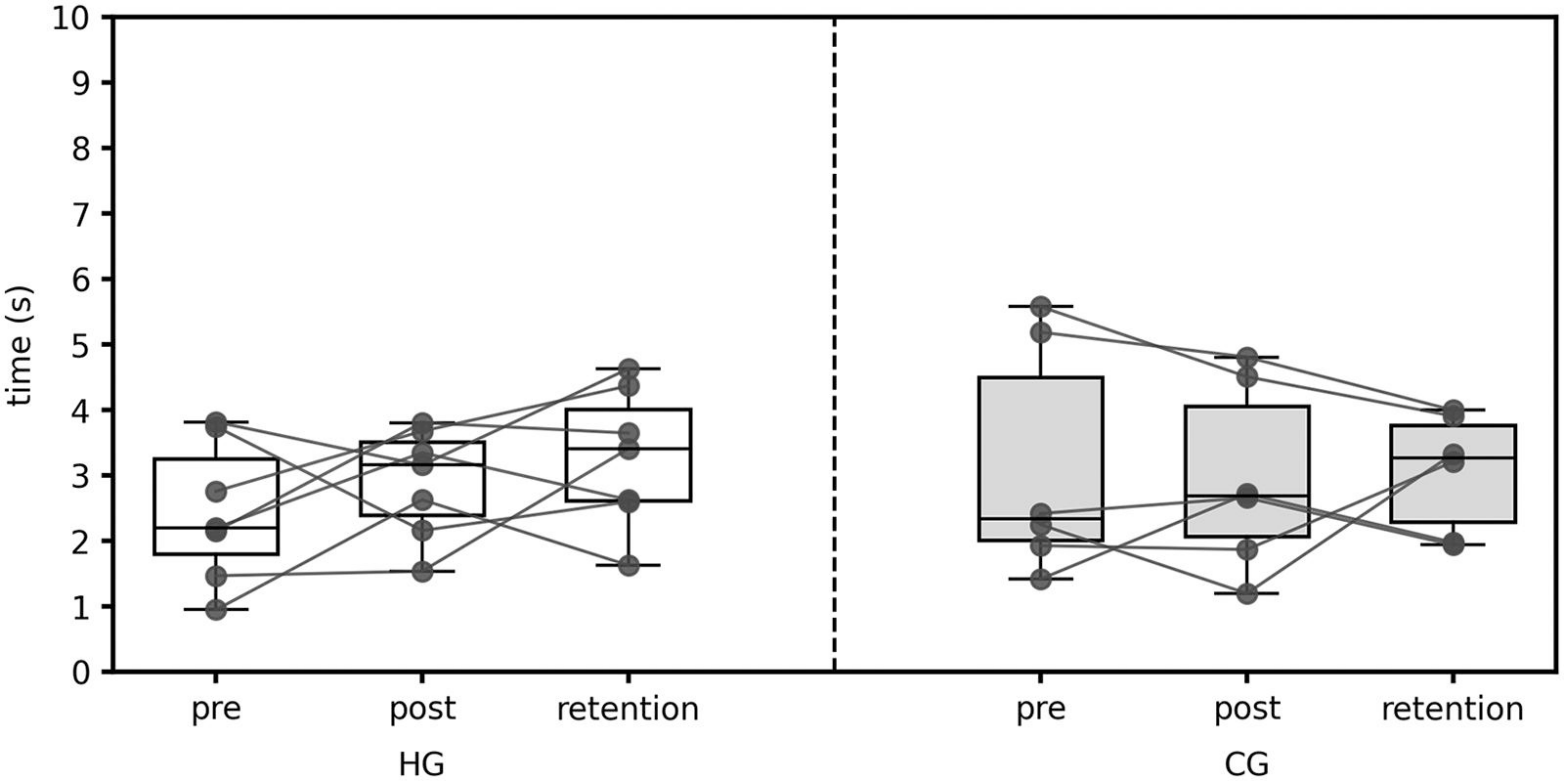
Box plots and intra-individual trends of mod. Romberg test with eyes closed at the measurement points (pre, post, and retention), separated by HG and CG. Note. s = seconds; horizontal line = median; box = interquartile range; dispersion of values via whiskers; intra-individual trends are shown by connecting the measured values per person at the three measurement points within the respective group.

Although the descriptive data from the modified Romberg test with eyes closed appeared to show group differences, these differences did not reach statistical significance. There was no significant interaction between measurement time and study group, F(2, 22) = 1.08, *p* = .358, partial *η*^2^ = .09. Additionally, there was no significant main effect of time in either the HG, *χ*^2^(2) = 3.71, *p* = .156, W = .27, or the CG, *χ*^2^(2) = 0.33, *p* = .846, *W* = .03 (Figure 3).

### 3.2 Dynamic steady-state balance

**Figure 4:**
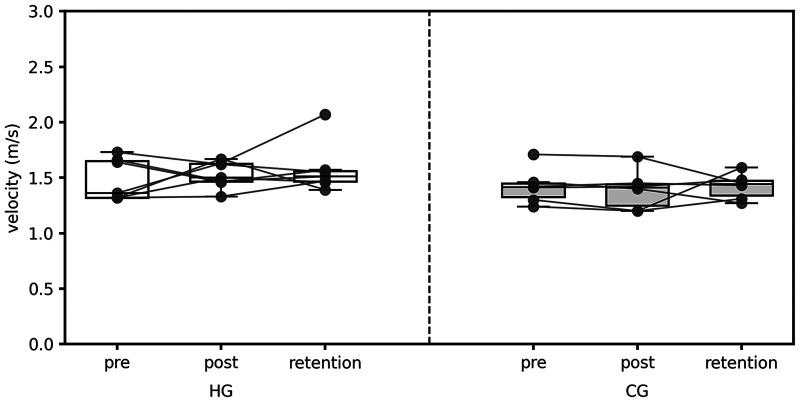
Box plots and intra-individual trends of 10 m walkway test at the measurement points (pre, post, and retention), separated by HG and CG. Note. m/s = meter/seconds; horizontal line = median; box = interquartile range; dispersion of values via whiskers; intra-individual trends are shown by connecting the measured values per person at the three measurement points within the respective group.

The data revealed no significant interaction on 10 m walkway performance between measurement time and study group was observed, F(2, 22) = 0.37, *p* = .694, partial *η*^2^ = .03. In addition, the test revealed no significant effects of time in either the HG, *χ*^2^(2) = 0.29, *p* = .867, *W* = .02, or the CG *χ*^2^(2) = 0.33, *p* = .846, *W* = .03 (Figure 4).

### 3.3 Proactive balance

**Figure 5:**
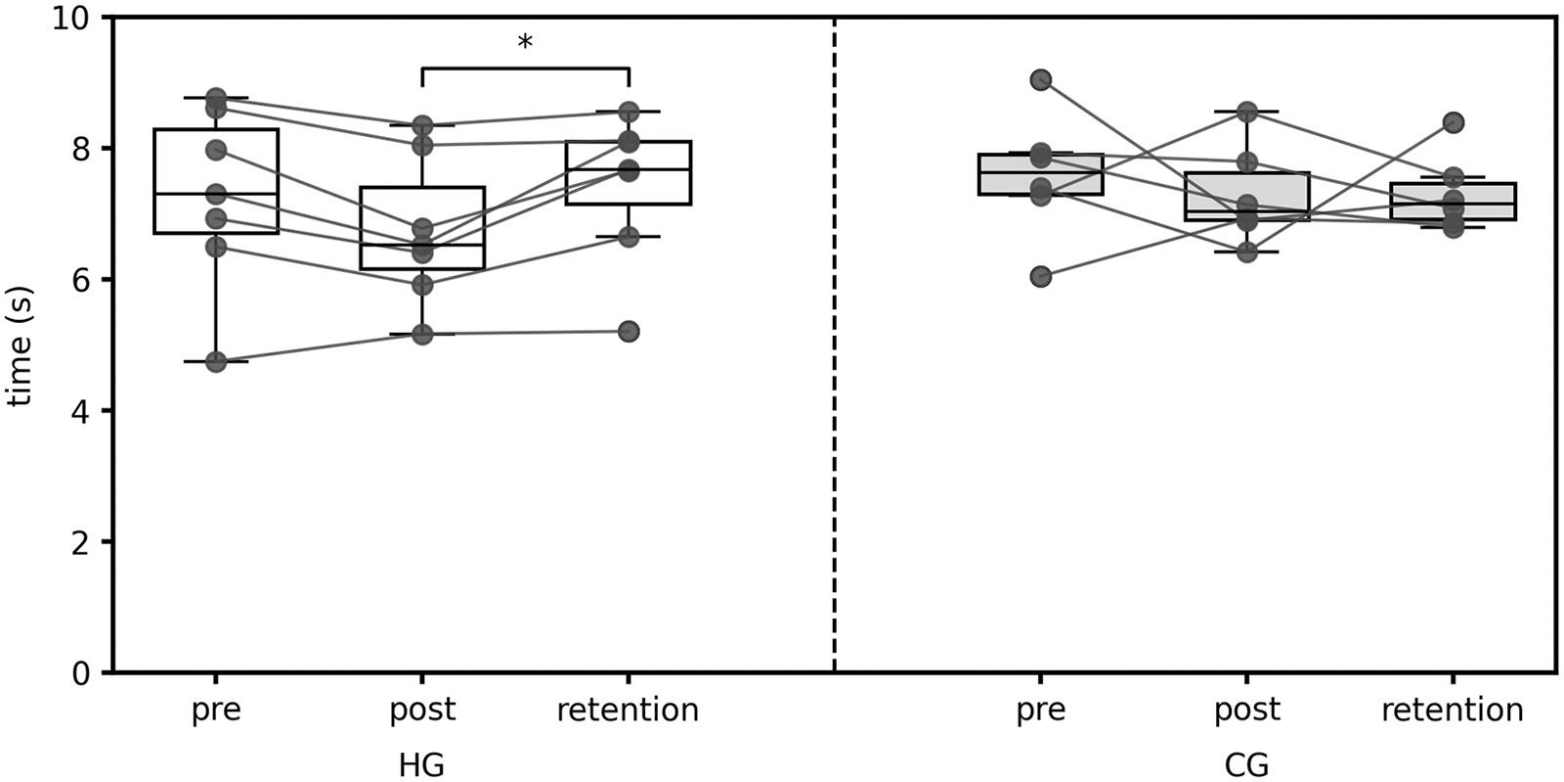
Box plots and intra-individual trends of timed up & go test at the measurement points (pre, post, and retention), separated by HG and CG. Note. s = seconds; horizontal line = median; box = interquartile range; dispersion of values via whiskers; intra-individual trends are shown by connecting the measured values per person at the three measurement points within the respective group; *p < .05.

While descriptive differences of the timed up & go test were observed, these differences were not supported by significant interaction between measurement time and study group, F(2, 22) = 0.93, *p* = .410, partial *η*^2^ = .08. A significant main effect of time was observed in the HG, *χ*^2^(2) = 8.00, *p* = .018, *W* = .57. post hoc pairwise comparisons revealed a significant difference between post- and retention-test (*z* = −1.43, *p* = .023, *r* = .54), indicating a large effect size according to Cohen (1988). No significant difference was found between pre- and post-test (*z* = −1.14, *p* = .098, *r* = .43) or pre- and retention-test (*z* = −0.29, *p* = 1.000, *r* = .11). There was no significant effect of time in the CG, *χ*^2^(2) = 0.33, *p* = .846, *W* = .03 (Figure 5).

### 3.4 Reactive balance

**Figure 6:**
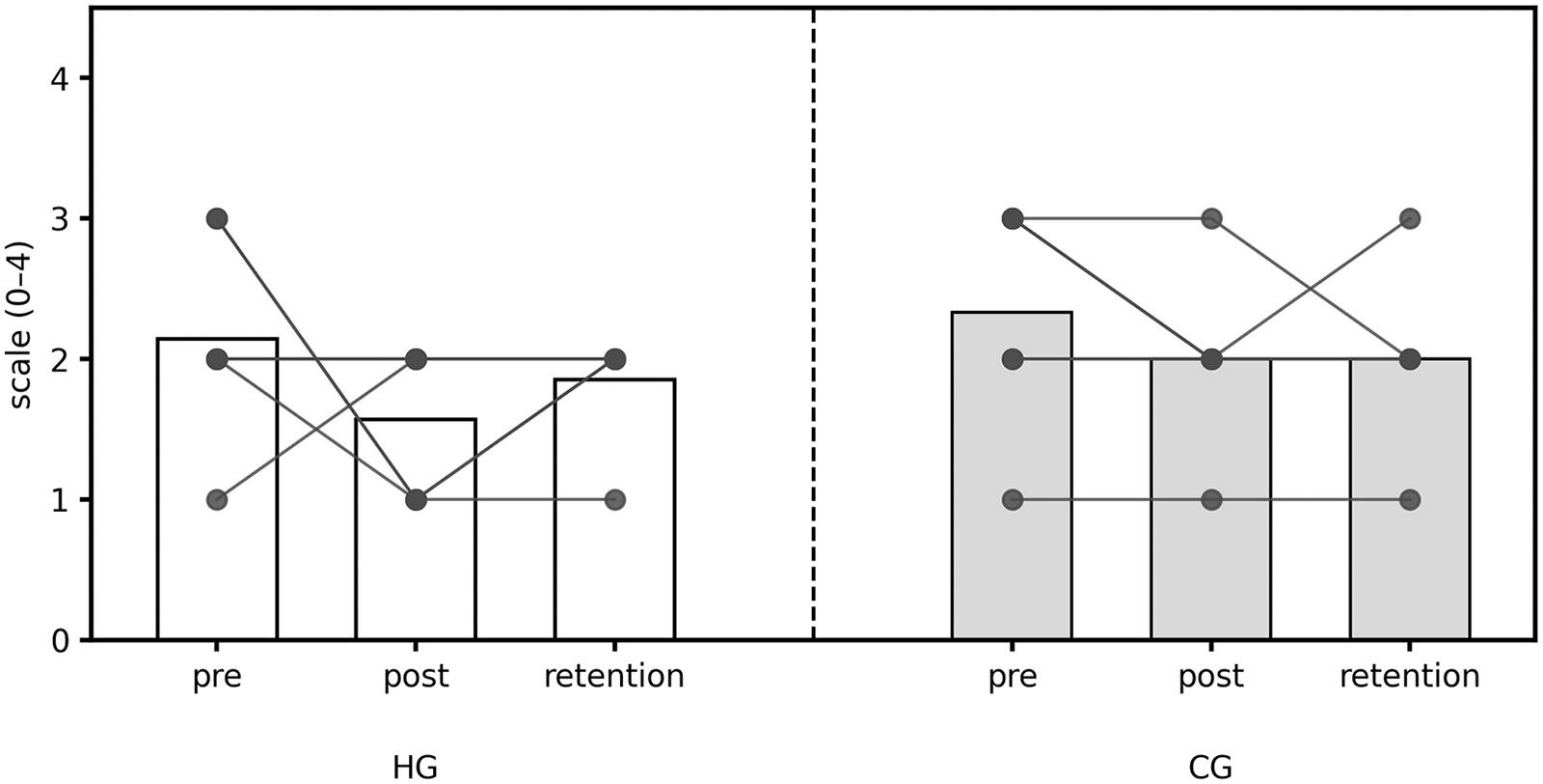
Bars and intra-individual trends of push & release test at the measurement points (pre, post, and retention), separated by HG and CG. Note. bars = mean; intra-individual trends are shown by connecting the measured values per person at the three measurement points within the respective group.

Between-group comparisons using rmANOVA were not conducted for the push & release test due to the ordinal nature of the outcome data. Thus, the differences in change scores between groups were examined using Mann–Whitney U tests (see Table 2 for descriptive statistics). No statistically significant differences were observed across any interval (*Δ*1: (*U* = 19.00, *z* = −.313, *p* = .754, *r* = .09), *Δ*2: (*U* = 16.00, *z* = −.880, *p* = .379, *r* = .24), and *Δ*3: (*U* = 20.00, *z* = −.155, *p* = .877, *r* = .04). No significant effect of time was observed in either the HG, *χ*^2^(2) = 2.29, *p* = .319, *W* = .16, or the CG, *χ*^2^(2) = 2.00, *p* = .368, *W* = .17 (Figure 6).

### 3.4 Leg strength

**Figure 7:**
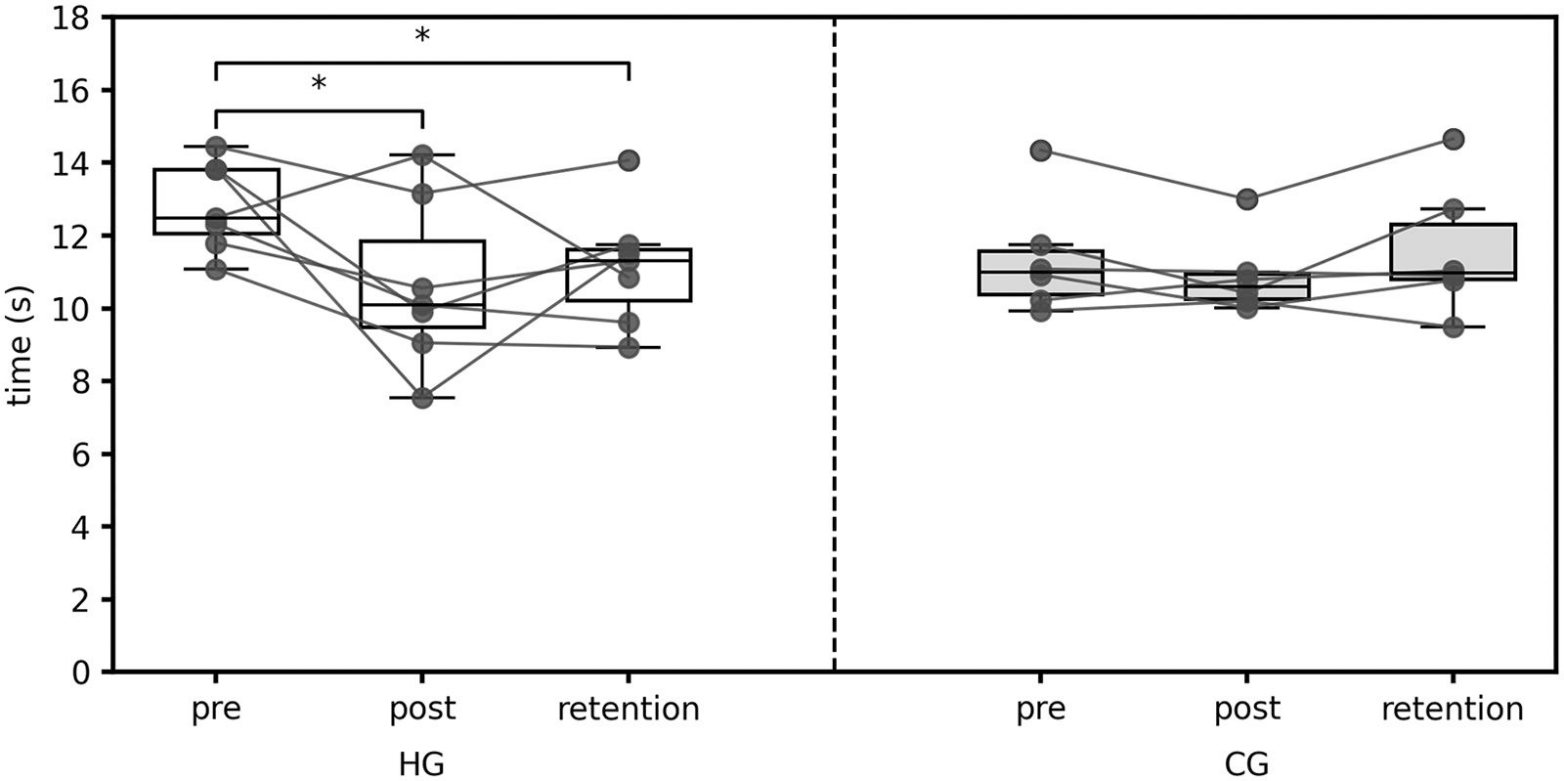
Box plots and intra-individual trends of chair rise test at the measurement points (pre, post, and retention), separated by HG and CG. Note. s = seconds; horizontal line = median; box = interquartile range; dispersion of values via whiskers; intra-individual trends are shown by connecting the measured values per person at the three measurement points within the respective group; *p < .05.

Although the descriptive results of the chair rise test seemed to indicate potential group differences, these were not statistically proven, as the interaction between measurement time and study group was not significant, F(1.26, 13.88) = 2.80, *p* = .111, partial *η*^2^ = .20. A significant main effect of time was observed in the HG, *χ*^2^(2) = 7.71, *p* = .021, *W* = .55. post hoc analyses revealed significant differences between pre- and post-test (*z* = −1.29, *p* = .048, *r* = .49) and between pre- and retention-test (*z* = −1.29, *p* = .048, *r* = .49), both reflecting a medium effect size according to Cohen (1988). No significant difference was found between post- and retention-test (*z* = 0.00, *p* = 1.000, *r* = .00). In the CG, no significant effect of time was observed, *χ*^2^(2) = 1.00, *p* = .607, *W* = .08 (Figure 7).

## 4 Discussion

This study examined the effects of small-sided team handball-based activities on balance and leg strength in inactive, healthy older adults. rmANOVA revealed no significant group × time interaction effects. However, complementary nonparametric between-group comparisons for variables in which the rmANOVA assumptions were violated showed a large, but not statistically significant, difference in change scores for static steady-state balance (eyes open) from pre- to post-test (*r* = .52), as well as a statistically significant difference from pre- to retention-test (*r* = .60). The HG demonstrated greater improvements than the CG. These findings suggest a delayed training effect, whereby initial static steady-state balance adaptations continued to develop or stabilize beyond the active intervention period. However, due to the absence of monitoring during the retention phase, this interpretation is considered tentative. Furthermore, no significant within-group differences were observed between measurement times for static steady-state, dynamic steady-state, and reactive balance variables. Due to the limited significant group effects, the findings only partially support the study hypothesis that the intervention would lead to improvements in balance and leg strength. While most balance parameters did not reach statistical significance, the small to moderate within-group effect sizes in HG (*W* = .14–.27) suggest the presence of potential functional adaptations. A significant within-group improvement in leg strength was observed in the HG; however, the absence of significant group × time interactions limits the ability to attribute this effect specifically to the intervention. The large effects for leg strength in HG (*W* = .55, *r* = .49) may indicate potentially meaningful changes, although these findings should be interpreted with caution given the limited statistical power. In addition, proactive balance declined from the post-intervention measurement to retention measurement with a large effect size (*W* = .57, *r* = .54). Importantly, the design of the present study does not allow for conclusions about handball-specific effects. Rather, it allows for conclusions about the potential impact of participating in a structured physical activity program compared to maintaining habitual activity levels.

### 4.1 Static steady-state balance

Between-group comparison revealed a large, but not statistically significant, difference in change scores for the modified Romberg test with eyes open from pre- to post-test (*r* = .52) and a statistically significant between-group difference from pre- to retention-test (*r* = .60) with HG demonstrating greater improvements than the CG. Although a descriptive analysis of the modified Romberg test with the eyes open or closed shows an increase in standing time in the HG, no significant within-group effect was observed throughout the study (Figures 2, 3). Previous studies have demonstrated the effectiveness of team handball-based activities on static steady-state balance (18, 37, 38). The positive effects of team sports on balance have also been observed in other sports (17, 38, 39). Studies on soccer-based activities have shown greater improvements in balance after the intervention period. This difference may be partly due to the longer duration (12–16 weeks) of those interventions (17, 38–40). Taking these aspects into account, it can be assumed that the duration of the team handball-based exercise covered in this study was too short to reveal significant within-group effects for this age category. The large between-group effect sizes (*r* = .52–.60) may also suggest a delayed training effect, wherein initial balance adaptations continued to develop or stabilize after the active intervention period ended. However, this interpretation is considered tentative due to the absence of monitoring during the retention phase. Similarly, the moderate within-group effect size (*W* = .27) indicates small but potentially meaningful improvements in balance performance that may not have reached statistical significance due to the limited sample size or study period. This interpretation aligns with studies showing that balance adaptations in older adults typically emerge after 10–12 weeks of consistent exposure (41). However, it is only possible to a limited extent to compare soccer- and team handball-based activities in static steady-state balance. Although the two sports have similar coordinative demands in terms of running, jumping, landing, stopping, and changing direction, soccer requires additional actions such as shooting and passing with the lower extremities, particularly on one leg. These unilateral demands may enhance proprioceptive feedback and postural control, explaining the larger effects observed in soccer-related studies. Since maintaining balance on one leg while shooting or passing requires additional coordination, it can be assumed that the conducted team handball exercise stimulates the musculoskeletal system more in the upper body, which may explain the better performance in static steady-state balance.

In summary, the between-group analysis aligns with previous studies' findings that team sports-based activities have positive effects on static steady-state balance. The large effect sizes in between-group analysis suggest that the handball-based intervention may lead to meaningful improvements in static steady-state balance compared to the control group. The absence of significant within-group effects is likely due to the brief intervention period and small sample size rather than a true lack of effect.

### 4.2 Dynamic steady-state balance

Dynamic steady-state balance, as measured by the 10 m walkway test, showed no significant effect from a team handball-based intervention (Figure 4). Current research indicates that balance improves through various forms of physical activity. Established exercise methods, such as strength training, coordination training, fitness training, and balance training, have been shown to positively affect dynamic steady-state balance following a movement intervention (13, 42, 43). Malka and Hantiu (43) attributed the improvement to the high level of coordination required in a gymnastics intervention with a strong focus on balance exercises. Unlike such balance-focused interventions, the present team handball activities did not include explicit gait or stability drills, which may explain the negligible within-group effect size (*W* = .02). The team handball-based activities, which combines high-intensity, intermittent exercise with changes of direction, start-stop movements, and finishes, also places high demands on participants' coordination (19, 44). However, these movements are primarily reactive and game-oriented rather than task-specific to walking speed, which could limit transfer to the 10 m walkway test. The coordination requirements are not the only relevant factor, as demonstrated by the improvement in lateral dynamic steady-state balance after monotonous walking training (45). It should be noted that dynamic steady-state balance is measured by walking speed, making monotonous walking training close to the test conditions. This highlights the task-specific nature of dynamic balance: improvements are most pronounced when the exercise closely mirrors the testing conditions. The improvements observed in this study could be related to the targeted exercise of the test conditions. Notably, although the descriptive data shows a slight tendency toward faster walking speed through team handball-based activities, no significant improvements in dynamic steady-state balance occurred. Due to the small effect size, this trend should be interpreted cautiously and is unlikely to reflect a meaningful intervention effect. Nevertheless, the aerobic and anaerobic requirements place significant demands on participants, requiring them to maintain balance in dynamic situations (44). However, these demands alone may not lead to measurable balance improvements without targeted postural control challenges. According to Sherrington et al. (46), balance training combined with gait exercises is the most effective way to reduce the risk of falls. Therefore, it is reasonable to assume that adding balance-improving exercises for older adults could positively impact dynamic steady-state balance and successful fall prevention during team handball activities. Future team handball-based interventions should integrate dynamic stability drills to complement sport-specific play.

### 4.3 Proactive balance

Performance in the proactive balance test declined during the retention phase (HG). The time required to complete the timed up & go test increased from the pre- to the retention-test (Figure 5). This deterioration was statistically significant and associated with large effect sizes. To the best of our knowledge, only one study has examined the influence of team sports-based activities on proactive balance. According to Pedersen et al. (47), timed up & go test performance remained stable following prolonged floorball training. This suggests that proactive balance and functional mobility in older adults may be maintained during continuous structured exercise but decline rapidly once exercise ceases. The large within-group effect size (*W* = .57) highlights that the observed decline was substantial and likely reflects a real deterioration in functional balance capacity rather than random variation. Since there are only a limited number of comparable studies on team sports, it is important to interpret and evaluate the results in the context of other sports as well. These studies confirm that structured balance and strength training can enhance proactive balance across age groups (42, 48–50). However, the results can only be compared to a limited extent due to the variety of exercise interventions and the small number of studies. Nevertheless, the present large effect size suggests that the timed up & go test is sensitive to changes even in short-term interventions. Similarly, proactive balance is primarily assessed in clinical practice to predict falls and the general functional performance of patients (51, 52). As timed up & go test performance is closely linked to fall risk, the observed deterioration could indicate a temporary decrease in functional resilience, despite occurring over a short period of time. However, given the small sample size of the present study, it is unclear how applicable the results are to the general population. Despite this limitation, the large effect size suggests robust intra-individual change, warranting further investigation in larger cohorts. In summary, the cause of the deterioration cannot be conclusively determined, so further research is needed.

### 4.4 Reactive balance

Reactive balance, as measured by the push & release test, showed no significant effect from a team handball-based intervention (Figure 6). The within-group effect size was small (*W* = .16), indicating that the intervention produced minimal measurable change in reactive balance. Limited research is available on reactive balance and its effects in team sports. However, sports science research has intensively focused on disturbance-based exercise and its positive effects on reactive balance and control (53–56). This type of exercise involves targeted exercises for provoked falls. Conversely, team handball-based activities are characterized by constantly changing conditions that allow for a closer connection to everyday conditions (19, 44). While these dynamic and unpredictable game situations theoretically challenge postural reactions, the absence of targeted perturbation stimuli likely limited adaptations in reactive balance control. In order to protect the participants, the game format applied in the study was adapted to a non-contact version, deliberately avoiding external stimuli caused by physical contact. However, a more physical format, allowing controlled physical contact, could potentially improve reactive balance in a similar way to disturbance-based interventions.

The high initial scores in the push & release test indicate good baseline balance. When initial performance is already close to optimal, the neuromuscular adaptations induced by exercise tend to stagnate. For this reason, a ceiling effect can be assumed. Sundstrup et al. (41) note that testing balance after an exercise intervention with younger participants yields better results than with older adults. They also explain that a longer-term intervention leads to comparable results in older adults (41). Consequently, adaptations in balance may occur over extended periods, though more sensitive test procedures are necessary to ensure reliable detection of even minor adaptations.

### 4.5 Leg strength

Leg strength, as measured by the chair rise test, showed a significant positive effect after the team handball-based intervention (Figure 4). These findings partially support the hypothesis at the within-group level. However, the lack of significant interaction effects prevents firm conclusions about the efficacy of the intervention. The large within-group effect size (*W* = .55, *r* = .49) underscores that the observed improvement was not only statistically significant but also practically meaningful. Although HG showed improvements over time, these changes cannot be attributed to the intervention with certainty due to the absence of significant group × time interactions (Figure 7). The chair rise test is a valid indicator of lower-extremity strength and functional power generation, both of which are key determinants of independence in older adults (26). These results align with existing literature on the effects of team sports activities on the musculoskeletal system. For example, a twelve-week team handball intervention improved grip strength in adult men (18). Similarly, Krustrup et al. (39) and Helge et al. (40) reported substantial improvements in leg strength after 12–16 weeks of recreational team sports, suggesting that intermittent game formats provide sufficient neuromuscular stimuli. Therefore, it can be assumed that the musculoskeletal system of HG improved in terms of increased muscle strength (26, 38). This study expands on previous research by demonstrating that leg muscles and their function in the chair rise test can benefit from team handball-based activities. Given the relatively short eight-week duration, the magnitude of improvement indicates rapid neuromuscular adaptation, likely driven by directional changes and accelerations. While Krustrup et al. (39) demonstrated improved muscle function and increased maximum dynamic muscle strength, Fristrup et al. (38) observed no changes in static or dynamic muscle strength, suspecting only stimulation of muscle mass and activation. The existing inconsistency may be explained by the load parameters. First, Andersen et al. (20) suspect minor differences in the musculoskeletal system due to low intensity loads during team sports activities. On the other hand, the present study shows that handball-based activities result in significant improvement despite an eight-week intervention period. These findings support the notion that even moderate intensity, game-based activities can meaningfully improve strength in previously inactive older adults, provided sufficient frequency and engagement. Given this, it is reasonable to assume that longer-term interventions will result in greater improvements in musculoskeletal parameters (15, 18, 20, 38, 40). Nonetheless, the present large effect already demonstrates that meaningful gains are achievable even within short interventions, emphasizing the responsiveness of older adults to well-designed, engaging exercise formats. Beyond leg strength alone, various studies have linked increased muscle strength to improved balance in older adults (42, 57–59). Improved muscle strength ensures rapid muscle power generation when an individual's position becomes unstable, which is necessary for balance compensation (60). Nevertheless, improving balance probably requires additional or stronger sensorimotor and proprioceptive components that are not explicitly addressed in this intervention, which could explain the lack of simultaneous gains in balance. While team handball-based activities reliably increased muscle strength, effects on balance could only be assumed. Nevertheless, the intervention shows positive practical tendencies that should be addressed in future studies. Neuromuscular effects may become apparent more quickly than coordinative effects.

### 4.6 Limitations

One limitation of the present study is its small sample size. Since this study was designed as a pilot study, a formal *a priori* power analysis was not conducted. The small sample size results in reduced statistical power. Therefore, null findings should not be interpreted as indicating the absence of an effect. Rather, they should be seen as reflecting the expected power constraints inherent to pilot research. Reported effect sizes should be considered preliminary estimates that inform the planning of adequately powered future studies. Another limitation is the absence of objective monitoring of the control group's habitual physical activity. Although participants in the control group were instructed to maintain their usual daily routines and were excluded from participating in additional structured sport, their activity levels were not objectively assessed, i.e., using accelerometers. Although all participants were physically inactive at baseline, uncontrolled changes in habitual activity during the intervention period cannot be entirely ruled out. Additionally, physical activity was not monitored during the six-week retention phase. Therefore, it is unclear whether changes in usual daily physical activity contributed to the observed between-group difference, suggesting that the findings regarding a delayed training effect should be interpreted with caution. The non-randomized quasi-experimental design and the self-selection of participants into groups cannot rule out systematic differences between the study groups because pre-existing group differences may confound the interpretation of intervention effects. For example, the HG was, on average, approximately six years older than the CG (72.57 ± 5.44 vs. 66.83 ± 2.64 years). This difference in age may have influenced baseline performance levels and the capacity to respond to the intervention. Given the small sample size and potential violations of normality, the results of the rmANOVA should be interpreted with caution because parametric tests may not be robust under these conditions. A potential experimenter bias cannot be excluded. Finally, the duration of the intervention must be considered. Evidence suggests that older adults often require longer intervention periods to elicit measurable neuromuscular and balance-related adaptations (41). The eight-week intervention used in this study may not have provided sufficient stimulus for more pronounced improvements in all outcome measures. Despite its limitations, this study was the first to examine the effects of a team handball-based intervention on balance and leg strength in older adults, providing valuable preliminary data. Future studies should increase the sample size and employ a randomized controlled trial design with an active control group that is matched for training volume and intensity. Additionally, physical activity levels should be objectively monitored throughout the intervention period and the retention phase.

### 4.7 Outlook

Given the results and limitations of this study, future research should examine the effects of team sports-based activities in older adults over longer intervention periods. Such studies could determine whether neuromuscular adaptations precede coordinative adaptations and whether improvements in balance become apparent only after prolonged exposure to the intervention. Although no significant group × time interactions between groups were found, complementary between-group comparisons revealed large effects for static steady-state balance (*r* = .52–.60). Within-group analysis also indicated a large effect for leg strength (*W* = .55), suggesting positive developments in the HG. Considering the significant between-group difference at the retention-test (*r* = .60) and the large effect at the post-test (*r* = .52), the possible delayed training effect warrants further investigation in adequately powered future studies. Significant effects, particularly with regard to balance, may only occur after prolonged exposure to stimuli (41). Based on the initial evidence of positive effects demonstrated in this study, a randomized controlled trial using standardized clinical or instrumental measurement methods is recommended. Future randomized controlled trials should integrate instrumented postural assessments (e.g., force plate, inertial sensors) as well as multidimensional balance assessments (e.g., Y-Balance Test, Functional Movement Screen) to increase sensitivity to subtle balance changes. Furthermore, balance testing, as emphasized by Granacher et al. (26), should be prioritized to investigate fall-prevention interventions in older adults. Additionally, older adults with more severe functional limitations should be examined. Current research confirms the feasibility and practicability of team sports-based activities with inactive, healthy older adults. Previous studies have confirmed a low injury rate in recreational team sports, indicating that these activities are safe for different target groups (15). Similarly, no injuries occurred during the team handball-based intervention in the present study. This also allows for further testing with groups more prone to falls and may benefit more significantly from team sports activities. Given the positive effects of team sports-based activities on the musculoskeletal, cardiovascular, and metabolic systems, it can be assumed that team sports promote health [e.g. (16–18, 37),]. The present study builds on these findings by examining factors that influence the risk of falls in older adults. Considering the functional and health-related aspects of team sports activities represents a promising approach for holistic strategies to prevent falls. However, future research should investigate the extent to which team sports-based activities have positive effects in older adults. Direct comparisons of different team sports activities are recommended. In this context, the present findings provide a preliminary basis for future studies to examine the role of team handball-based and other team sports-based activities in fall prevention programs for older adults.

### 4.8 Conclusion

Between-group analysis revealed large effects for static steady-state balance, suggesting a possible delayed training effect. Leg strength increased significantly after the small-sided team handball-based intervention. These findings suggest that team handball-based activities may provide a beneficial neuromuscular stimulus. As one of the first studies to examine task-specific balance outcomes in team sports activities, this study offers preliminary insights into the potential and limitations of these exercise methods. Overall, the results underscore the health-promoting potential of team sports activities for older adults, particularly with regard to static steady-state balance and musculoskeletal function. Further research with larger samples and extended intervention periods is needed to clarify whether handball-based interventions can improve balance-related parameters and contribute to fall prevention.

## Data Availability

The raw data supporting the conclusions of this article will be made available by the corresponding author without undue reservation.
